# Supplementary material for: Many-body simulation of two-dimensional electronic spectroscopy of excitons and trions in monolayer transition metal dichalcogenides
Source: Nat Commun. 2019 Jul 31;10:3419. doi: 10.1038/s41467-019-11497-y (PMC6668418; doi:10.1038/s41467-019-11497-y)
Supplement: Supplementary file 1 — Supplementary Information [file 41467_2019_11497_MOESM1_ESM.pdf]

Supplementary Information for "Many-body simulation of two-dimensional electronic spectroscopy of excitons and trions in monolayer transition metal dichalcogenides"

Roel Tempelaar*,[1] and Timothy C. Berkelbach†,[1,2]

[1]Department of Chemistry, Columbia University, 3000 Broadway, New York, New York 10027, USA.

[2]Center for Computational Quantum Physics, Flatiron Institute, New York, New York 10010 USA.

*r.tempelaar@gmail.com

†tim.berkelbach@gmail.com

S1 Supplementary Methods

S1.1 Band structure and optical properties

Monolayer transition metal dichalcogenides (TMDCs) are known to exhibit a direct band gap, with valence band maxima and conduction band minima located at the K and K' points, which occur at alternating corners of the hexagonal first Brillouin zone. When compared to first-principles calculations, previous works have shown the quasiparticle band structure in the vicinity of these high-symmetry points to be well approximated by a gapped massive Dirac-like two-band Hamiltonian,[1–3] given by

$$H(\mathbf{k}) = \begin{pmatrix} -\epsilon + \lambda_{\mathrm{v}}\tau_{\mathbf{k}}L_z & at(\tau_{\mathbf{k}}\bar{k}_x + i\bar{k}_y) \\ at(\tau_{\mathbf{k}}\bar{k}_x - i\bar{k}_y) & \epsilon + \lambda_{\mathrm{c}}\tau_{\mathbf{k}}L_z \end{pmatrix}. \tag{S1}$$

Here, a is the lattice constant, t the effective nearest-neighbor transfer integral, and $2\epsilon \equiv E_{\mathrm{g}}$ is related to the material band gap. The Hamiltonian includes valley-dependent spin-orbit splitting of the valence (v) and conduction (c) bands, quantified by $2\lambda_{\mathrm{v/c}}$, with the Pauli operator for spin, L_z, returning $+1$ and -1 for spin-up ($\uparrow$) and spin-down ($\downarrow$) quasiparticles, respectively. Values of the applied parameters are summarized in Tab. S1.

The two-band Hamiltonian given in Eq. S1 is linearized in $\bar{\mathbf{k}}$, the wavevector relative to the nearest K or K' point, that is, $\bar{\mathbf{k}} = \mathbf{k} - \mathbf{K}$ or $\bar{\mathbf{k}} = \mathbf{k} - \mathbf{K}'$ (whichever is smaller). The two "valleys" surrounding these points are indexed by $\tau_{\mathbf{k}} = \pm 1$. The spinor basis in which the Hamiltonian is expressed corresponds to the transition metal $|d_{z^2}\rangle \equiv |\phi^{\mathrm{c}}\rangle$ orbital and the symmetry adapted $|d_{x^2-y^2}\rangle + i\tau_{\mathbf{k}}|d_{xy}\rangle \equiv |\phi^{\mathrm{v}}_{\tau_{\mathbf{k}}}\rangle$ orbital. With both spin states completely decoupled (so that L_z remains a good quantum number), the associated eigenvalue equation takes the form

$$H(\mathbf{k})\,|\psi^{\mathrm{v/c}}_{\mathbf{k},\sigma}\rangle = E^{\mathrm{v/c}}_{\mathbf{k},\sigma}\,|\psi^{\mathrm{v/c}}_{\mathbf{k},\sigma}\rangle\,, \tag{S2}$$

where $\sigma = \uparrow, \downarrow$ labels spin.

A great advantage of the two-band model is that it can be solved analytically, yielding simple expressions for the single-particle optical selection rules, which are found to be largely

Parameter	Symbol	MoS_2	$MoSe_2$	WS_2	WSe_2
Band gap	E_{g}	1.66 eV	1.47 eV	1.79 eV	1.60 eV
Spin-orbit splitting					
valence	λ_{v}	148 meV	184 meV	430 meV	466 meV
conduction	λ_{c}	-3.0 meV	-31.0 meV	26.0 meV	36.0 meV
Transfer integral	t	1.10 eV	0.94 eV	1.37 eV	1.19 eV
Lattice constant	a	3.19 Å	3.31 Å	3.20 Å	3.31 Å
2D polarizability	χ_{2D}	6.60 Å	8.23 Å	6.03 Å	7.18 Å

Table S1: Model parameters for the different TMDCs studied in our work.

conserved even in the presence of electron-hole interactions.[3] This advantage notwithstanding, we here employ the two-band model merely as a physically insightful means to obtain a faithful reproduction of first-principles calculated bandstructures near the K and K' points at arbitrary (and *in principle* infinite) samplings of the Brillouin zone. This, in turn, allows us to accurately evaluate optical transitions near the band edge, whose dominant contributions almost exclusively originate from these select Brillouin zone regions (see main text). This principle was also employed in Ref. 3, where calculations of band-edge exciton absorption spectra showed negligible differences between the two-band model and an extended model based on three bands.

The optical properties of a material are ultimately determined by the momentum matrix elements between the single-particle valence and conduction states, defined as

$$\mathbf{P}^{\mathrm{vc}}(\mathbf{k},\sigma) = -\frac{im}{\hbar} \langle \psi^{\mathrm{v}}_{\mathbf{k},\sigma} | \, [\mathbf{r}, H(\mathbf{k})] \, | \psi^{\mathrm{c}}_{\mathbf{k},\sigma} \rangle \, , \tag{S3}$$

where m is the free-electron mass and $\mathbf{r} = i\nabla_{\mathbf{k}}$ the position operator. In monolayer TMDCs, the out-of-plane component of these elements are zero by symmetry. Moreover, a remarkable feature attributed to monolayer TMDCs is that valley-selective excitation near the K and K' points can be realized by use of circularly polarized light. The optical selection rules under such polarization conditions are determined by the combination

$$P^{\mathrm{vc}}_{\pm}(\mathbf{k},\sigma) = \frac{1}{\sqrt{2}}[P^{\mathrm{vc}}_{x}(\mathbf{k},\sigma) \pm iP^{\mathrm{vc}}_{y}(\mathbf{k},\sigma)]. \tag{S4}$$

Upon analytically solving the two-band model, this can be shown to take the form[1]

$$P^{\mathrm{vc}}_{\pm}(\mathbf{k},\sigma) = \frac{mat}{\hbar}\Big(1 \pm \tau_{\mathbf{k}} \frac{E_{\mathrm{g}}}{E^{\mathrm{c}}_{\mathbf{k},\sigma} - E^{\mathrm{v}}_{\mathbf{k},\sigma}}\Big), \tag{S5}$$

from which the valley helicity selectiveness becomes readily obvious.

S1.2 Light absorbing excitons

In the undoped limit, light absorption promotes an electron from the fully occupied valence band to the unoccupied conduction band, yielding exciton states of the form

$$|\Psi^{\alpha}\rangle = \sum_{\boldsymbol{\kappa}} A^{\alpha}_{\boldsymbol{\kappa}} c^{\dagger}_{\boldsymbol{\kappa}} b_{\boldsymbol{\kappa}} |0\rangle \, , \tag{S6}$$

with α labeling the states. For notational convenience, we have introduced a generalized index $\boldsymbol{\kappa}$ labeling both momentum and spin state, i.e., $\boldsymbol{\kappa} = (\mathbf{k},\sigma)$. Annihilation of a valence band electron and creation of a conduction band electron is represented by the operators $b_{\boldsymbol{\kappa}}$ and $c^{\dagger}_{\boldsymbol{\kappa}}$, respectively, acting on the vacuum state $|0\rangle$, while it is understood that the involved light field carries negligible momentum and does conserve spin. These operators relate to those used in Eq. 1 of the main text by the relations $b^{(\dagger)}_{\boldsymbol{\kappa}} = a^{(\dagger)}_{\mathrm{v}\sigma,\mathbf{k}}$ and $c^{(\dagger)}_{\boldsymbol{\kappa}} = a^{(\dagger)}_{\mathrm{c}\sigma,\mathbf{k}}$, where the subscripts v$\sigma$ and cσ refer to the valence and conduction bands with spin σ, respectively.

An effective model to account for the exciton coefficients $A^{\alpha}_{\boldsymbol{\kappa}}$ is provided by the Bethe-Salpeter equation (BSE) formalism, which takes the form of an eigenvalue equation for the exciton energy E^{α},

$$E^{\alpha} A^{\alpha}_{\boldsymbol{\kappa}} = (E^{\mathrm{c}}_{\boldsymbol{\kappa}} - E^{\mathrm{v}}_{\boldsymbol{\kappa}}) A^{\alpha}_{\boldsymbol{\kappa}} + \frac{1}{A} \sum_{\mathbf{q}} \langle \psi^{\mathrm{v}\dagger}_{\boldsymbol{\kappa}} \psi^{\mathrm{c}}_{\boldsymbol{\kappa}} | \, K^{\mathrm{int}} \, | \psi^{\mathrm{v}\dagger}_{\boldsymbol{\kappa}+\mathbf{q}} \psi^{\mathrm{c}}_{\boldsymbol{\kappa}+\mathbf{q}} \rangle \, A^{\alpha}_{\boldsymbol{\kappa}+\mathbf{q}}, \tag{S7}$$

where K^{int} represents the quasiparticle interaction kernel, the summation of which is normalized to the total Brillouin zone area, A. The BSE implicitly assumes this kernel to conserve the total momentum of the electron-hole pair, being formulated in the subspace in which the electron momentum minus the hole momentum is conserved and equal to zero. In addition, K^{int} conserves the spin of individual particles, so that $\boldsymbol{\kappa}+\mathbf{q}$ refers to $(\mathbf{k}+\mathbf{q}, \sigma)$, as a result of which the BSE takes a block diagonal form with respect to σ. Importantly, when considering energy and helicity selective excitation of a spin-up electron at the K valley (as done in the main text), only the block with $\sigma = \uparrow$ needs evaluation.

The daggers appearing in the quasiparticle interaction kernel refer to hole particle states (which are Hermitian conjugates of electron particle states). Following the example of Ref. 3, we neglect exchange interaction between conduction and valence band electrons, and approximate this kernel as

$$\langle \psi^{\mathrm{v}\dagger}_{\boldsymbol{\kappa}} \psi^{\mathrm{c}}_{\boldsymbol{\kappa}} | \, K^{\mathrm{int}} \, | \psi^{\mathrm{v}\dagger}_{\boldsymbol{\kappa}+\mathbf{q}} \psi^{\mathrm{c}}_{\boldsymbol{\kappa}+\mathbf{q}} \rangle \approx - \langle \psi^{\mathrm{v}}_{\boldsymbol{\kappa}+\mathbf{q}} | \psi^{\mathrm{v}}_{\boldsymbol{\kappa}} \rangle \, \langle \psi^{\mathrm{c}}_{\boldsymbol{\kappa}} | \psi^{\mathrm{c}}_{\boldsymbol{\kappa}+\mathbf{q}} \rangle \, W(\mathbf{q}), \tag{S8}$$

with the quasiparticle wavevectors from Eq. S2, and with the screened Coulomb interaction term given by[4,5]

$$W(\mathbf{q}) = \frac{2\pi e^2}{q(1 + 2\pi \chi_{\mathrm{2D}} q)}. \tag{S9}$$

Here, χ_{2D} denotes the two-dimensional polarizability, which is readily parametrized against first-principles calculations[6] (see Tab. S1 for parameters used in this work).

S1.3 Light absorbing singlet and triplet trions

Negatively-charged trions, in which two electrons interact with one hole, result when light absorption creates an electron-hole pair in an n-doped material. The BSE for excitons can straightforwardly be generalized to describe negatively charged trions with pairwise interactions. Without imposing spin and momentum restrictions on the participating quasiparticles, and again neglecting exchange interactions between conduction and valence band electrons,

the resulting equation reads[2,7,8]

$$
\begin{aligned}
E^{\alpha} B^{\alpha}_{\kappa_1,\kappa_2,\kappa_3} = {} & (E^{\mathrm{c}}_{\kappa_2} + E^{\mathrm{c}}_{\kappa_3} - E^{\mathrm{v}}_{\kappa_1}) B^{\alpha}_{\kappa_1,\kappa_2,\kappa_3} \\
& - \frac{1}{A} \sum_{\mathbf{q}} \langle \psi^{\mathrm{c}}_{\kappa_1} | \psi^{\mathrm{c}}_{\kappa_1+\mathbf{q}} \rangle \langle \psi^{\mathrm{v}}_{\kappa_3+\mathbf{q}} | \psi^{\mathrm{v}}_{\kappa_3} \rangle W(\mathbf{q}) B^{\alpha}_{\kappa_1+\mathbf{q},\kappa_2,\kappa_3+\mathbf{q}} \\
& + \frac{1}{A} \sum_{\mathbf{q}} \langle \psi^{\mathrm{c}}_{\kappa_1} | \psi^{\mathrm{c}}_{\kappa_1+\mathbf{q}} \rangle \langle \psi^{\mathrm{v}}_{\kappa_3+\mathbf{q}} | \psi^{\mathrm{v}}_{\kappa_3} \rangle W(\mathbf{q}) B^{\alpha}_{\kappa_2,\kappa_1+\mathbf{q},\kappa_3+\mathbf{q}} \\
& - \frac{1}{A} \sum_{\mathbf{q}} \langle \psi^{\mathrm{c}}_{\kappa_2} | \psi^{\mathrm{c}}_{\kappa_2+\mathbf{q}} \rangle \langle \psi^{\mathrm{v}}_{\kappa_3+\mathbf{q}} | \psi^{\mathrm{v}}_{\kappa_3} \rangle W(\mathbf{q}) B^{\alpha}_{\kappa_1,\kappa_2+\mathbf{q},\kappa_3+\mathbf{q}} \\
& + \frac{1}{A} \sum_{\mathbf{q}} \langle \psi^{\mathrm{c}}_{\kappa_2} | \psi^{\mathrm{c}}_{\kappa_2+\mathbf{q}} \rangle \langle \psi^{\mathrm{v}}_{\kappa_3+\mathbf{q}} | \psi^{\mathrm{v}}_{\kappa_3} \rangle W(\mathbf{q}) B^{\alpha}_{\kappa_2+\mathbf{q},\kappa_1,\kappa_3+\mathbf{q}} \\
& + \frac{1}{A} \sum_{\mathbf{q}} \langle \psi^{\mathrm{c}}_{\kappa_1} | \psi^{\mathrm{c}}_{\kappa_1+\mathbf{q}} \rangle \langle \psi^{\mathrm{c}}_{\kappa_2} | \psi^{\mathrm{c}}_{\kappa_2-\mathbf{q}} \rangle W(\mathbf{q}) B^{\alpha}_{\kappa_1+\mathbf{q},\kappa_2-\mathbf{q},\kappa_3} \\
& - \frac{1}{A} \sum_{\mathbf{q}} \langle \psi^{\mathrm{c}}_{\kappa_1} | \psi^{\mathrm{c}}_{\kappa_1+\mathbf{q}} \rangle \langle \psi^{\mathrm{c}}_{\kappa_2} | \psi^{\mathrm{c}}_{\kappa_2-\mathbf{q}} \rangle W(\mathbf{q}) B^{\alpha}_{\kappa_2-\mathbf{q},\kappa_1+\mathbf{q},\kappa_3} .
\end{aligned}
\tag{S10}
$$

with the coordinates κ_1 and κ_2 representing the electrons, and κ_3 representing the hole.

Similarly to the exciton case, the trion BSE conserves the spin of individual quasiparticles as well as their total momentum, $\mathbf{k}_1 + \mathbf{k}_2 - \mathbf{k}_3$. Furthermore, in the resulting block diagonal form, only certain blocks are of interest depending on the physical situation at hand. An important distinction between such situations is based on the composite spin of the two electrons involved in the trion state, which is singlet or triplet depending on whether the individual electron spins are opposite or equal, respectively. The singlet trion states can be expanded as

$$
|\Psi^{\alpha}\rangle_{(\mathrm{S})} = \sum_{\mathbf{k}_1,\mathbf{k}_2,\mathbf{k}_3}{}' B^{\alpha}_{(\mathbf{k}_1,\downarrow),(\mathbf{k}_2,\uparrow),(\mathbf{k}_3,\uparrow)} c^{\dagger}_{\mathbf{k}_1,\downarrow} c^{\dagger}_{\mathbf{k}_2,\uparrow} b_{\mathbf{k}_3,\uparrow} |0\rangle , \tag{S11}
$$

where the prime on the summation refers to the momentum conservation constraint $\mathbf{k}_1 + \mathbf{k}_2 - \mathbf{k}_3 = \mathbf{Q}$, with $\mathbf{Q}$ as the total trion momentum. For optically prepared trion states, this momentum equals that of the initial single-electron state, which is expressed as

$$
|\Psi^{\mathrm{i}}\rangle_{(\mathrm{S})} = c^{\dagger}_{\mathbf{Q},\downarrow} |0\rangle . \tag{S12}
$$

At low temperature, $\mathbf{Q}$ is the global spin-down conduction band minimum, located at the K' point for MoX_2 and at the K point for WX_2, as discussed in the main text.

Analogously, the triplet trion states are expanded as

$$
|\Psi^{\alpha}\rangle_{(\mathrm{T})} = \sum_{\mathbf{k}_1>\mathbf{k}_2;\mathbf{k}_3}{}' B^{\alpha}_{(\mathbf{k}_1,\uparrow),(\mathbf{k}_2,\uparrow),(\mathbf{k}_3,\uparrow)} c^{\dagger}_{\mathbf{k}_1,\uparrow} c^{\dagger}_{\mathbf{k}_2,\uparrow} b_{\mathbf{k}_3,\uparrow} |0\rangle , \tag{S13}
$$

and the associated initial one-electron state as

$$|\Psi^{\mathrm{i}}\rangle_{(\mathrm{T})} = c^{\dagger}_{\mathbf{Q},\uparrow} |0\rangle \,. \tag{S14}$$

Conversely to the singlet case, $\mathbf{Q}$ at low temperature refers to the K point for MoX_2 and to the K' point for WX_2. Importantly, in Eq. S13 the summation is limited to $\mathbf{k}_1 > \mathbf{k}_2$ in order to avoid double counting of configurations of electrons, which are indistinguishable particles. This forms a marked contrast to the singlet trion states where electron states differ by spin. Note that for notational simplicity, in Eq. 1 of the main text the trion states are expanded in an alternative form, with the coefficients related to the above expansions as $C^{\alpha}_{\mathrm{c}\sigma_1,\mathrm{c}\sigma_2,\mathrm{v}\sigma_3}(\mathbf{k}_1,\mathbf{k}_2) = B^{\alpha}_{(\sigma_1,\mathbf{k}_1),(\sigma_1,\mathbf{k}_2),(\sigma_3,\mathbf{k}_1+\mathbf{k}_2)}$.

S1.4 Optical response

Linear absorption is accounted for by Fermi's Golden Rule, given in Eq. 2 of the main text. For exciton absorption, the initial state appearing in this equation is simply the vacuum state, $|\Psi^{\mathrm{i}}\rangle = |0\rangle$, with associated energy $E^{\mathrm{i}} = 0$, while the summation extends over the final states $|\Psi^{\alpha}\rangle$ given in Eq. S6. Restricting ourselves to energy and helicity selective optical excitation of spin-up electrons, and taking the incident light direction to be normal to the TMDC monolayer, the exciton absorption spectrum can be expressed as

$$S(\omega) = \frac{4\pi^2 e^2}{m^2\omega^2} \sum_{\alpha} |\sum_{\mathbf{k}} A^{\alpha}_{(\mathbf{k},\uparrow)} P^{\mathrm{vc}}_{-}(\mathbf{k},\uparrow)|^2 \Gamma(E^{\alpha} - E^{\mathrm{i}} - \hbar\omega), \tag{S15}$$

with $P^{\mathrm{vc}}_{-}(\mathbf{k},\uparrow)$ representing the momentum matrix elements from Eq. S4.

For trions, the initial and final states are given in Eqs. S11–S14, whereas the initial energies are given by $E^{\mathrm{i}} = E^{\mathrm{c}}_{\mathbf{Q}}$. This leaves Eq. S15 unaltered, safe for the replacement

$$A^{\alpha}_{(\mathbf{k},\uparrow)} \leftarrow B^{\alpha}_{(\mathbf{Q},\uparrow),(\mathbf{k},\uparrow),(\mathbf{k},\downarrow)} \tag{S16}$$

for singlet trions, whereas for triplet trions

$$A^{\alpha}_{(\mathbf{k},\uparrow)} \leftarrow H(\mathbf{Q}-\mathbf{k}) B^{\alpha}_{(\mathbf{Q},\uparrow),(\mathbf{k},\uparrow),(\mathbf{k},\uparrow)} - H(\mathbf{k}-\mathbf{Q}) B^{\alpha}_{(\mathbf{k},\uparrow),(\mathbf{Q},\uparrow),(\mathbf{k},\uparrow)} \tag{S17}$$

with the effects of exchange of electrons accounted for by means of the Heaviside step function H. Contained in these replacements is that light couples exclusively to electron-hole pairs with total momentum zero.

The expressions for two-dimensional electronic spectroscopy follow analogously, starting from the generalization of Fermi's Golden Rule given in Eq. 3 of the main text.

S1.5 Brillouin zone sampling and truncation

In our calculations, the exciton and trion BSEs are evaluated by representing the two-dimensional Brillouin zone by a discretized k-mesh, composed as a Monkhorst-Pack grid with

$N \times N$ points. The applied Brillouin zone truncation scheme involves a truncation radius, denoted k_0 in the main text, around the K and K' points. Specifically, when considering helicity-selective excitation of a spin-up exciton in the K valley, exciton basis states are restricted to those having coefficients $A^{\alpha}_{\mathbf{k},\uparrow}$ with $|\mathbf{k} - \mathbf{K}| < k_0$. For the trion states, each quasiparticle is analogously restricted to its respective valley, as illustrated in Fig. 1 of the main text. For a given pair of N and k_0, the resulting trion and exciton spectra are rigidly shifted such that the band-edge exciton state lies at 2.00 eV for sulphur-based TMDCs, and at 1.66 eV for selenium-based TMDCs. Although these shifts generally depend on the applied truncation parameters, typical values are on the order of 0.50–0.75 eV.

S2 Supplementary Figures

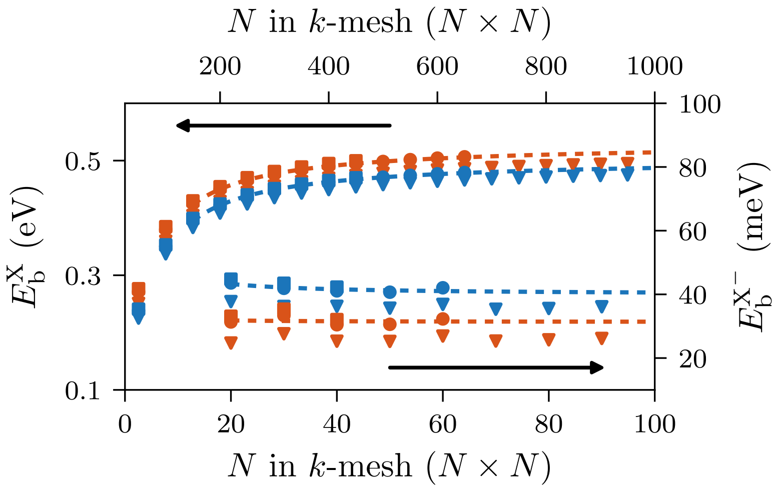

Supplementary Figure 1. Binding energies for sulphur-based TMDCs. Calculated exciton (X) and trion (X^-) binding energies for MoS_2 (red) and WS_2 (blue) are shown as a function of the k-mesh grid resolution N, and for Brillouin zone truncation radii of 0.10 (triangles), 0.16 (circles), and 0.20 (squares), in units of $2\pi/a$. Dashed curves represent fittings of $C_1 + C_2/N$ to the $k_0 = 0.16$ results from which the binding energies (C_1) reported in the main text are derived.

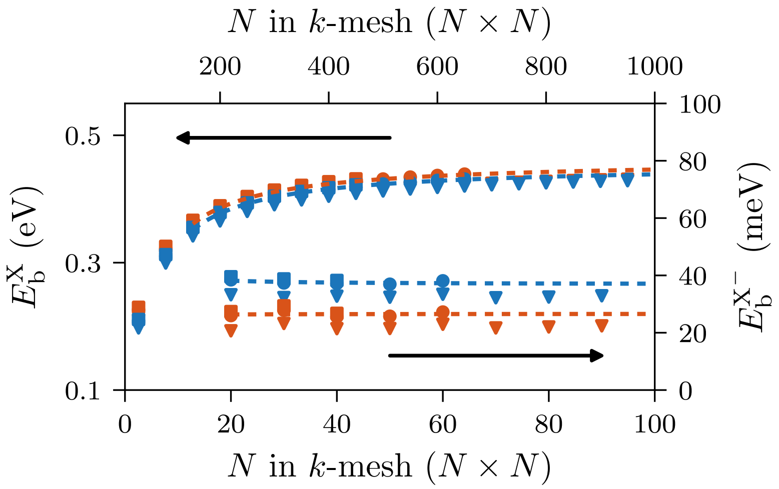

Supplementary Figure 2. Binding energies for selenium-based TMDCs. Same as Supplementary Fig. 1, but for $MoSe_2$ (red) and WSe_2 (blue).

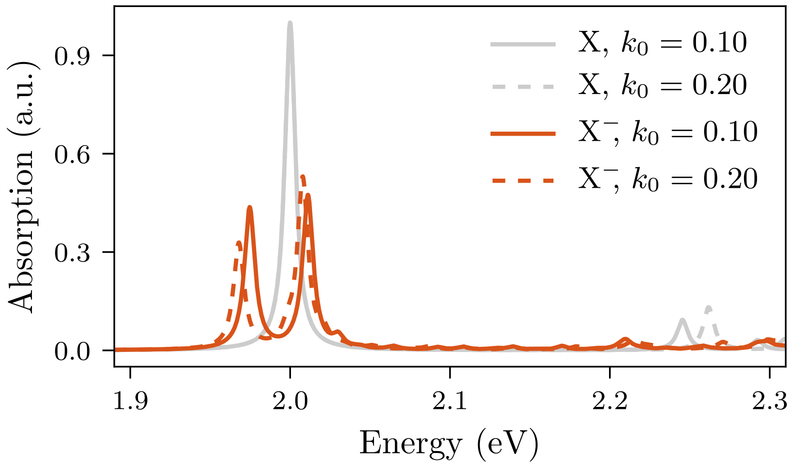

Supplementary Figure 3. Convergence of exciton and trion linear absorption with k_0. Shown are the exciton and singlet trion spectra for MoS_2 with $N = 40$.

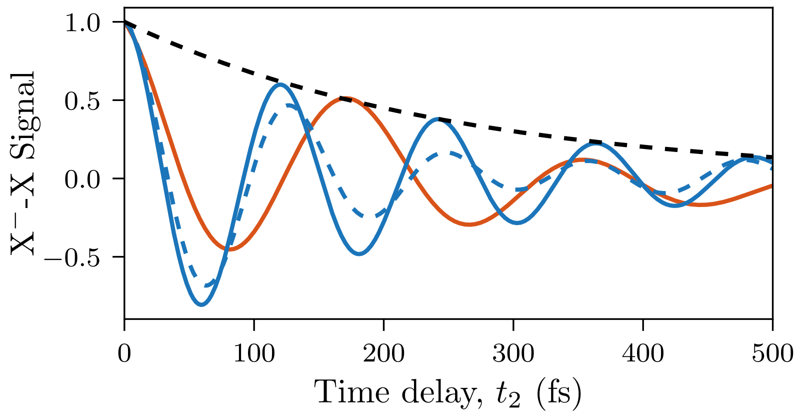

Supplementary Figure 4. Quantum beats at the upper cross-peak (X$^-$-X) location. Shown is the time-dependent signal for $MoSe_2$ (red), and for WSe_2 excluding (blue solid) and including (blue dashed) a non-degeneracy between the singlet and triplet trions. Shown as a reference is an exponential indicating the phenomenological exciton-trion coherence decay (black dashed).

Supplementary References

[1] Xiao, D., Liu, G.-B., Feng, W., Xu, X. & Yao, W. Coupled spin and valley physics in monolayers of MoS_2 and other group-VI dichalcogenides. *Phys. Rev. Lett.* **108**, 196802 (2012).

[2] Zhang, C., Wang, H., Chan, W., Manolatou, C. & Rana, F. Absorption of light by excitons and trions in monolayers of metal dichalcogenide MoS_2: Experiments and theory. *Phys. Rev. B* **89**, 205436 (2014).

[3] Berkelbach, T. C., Hybertsen, M. S. & Reichman, D. R. Bright and dark singlet excitons via linear and two-photon spectroscopy in monolayer transition-metal dichalcogenides. *Phys. Rev. B* **92**, 085413 (2015).

[4] Berkelbach, T. C., Hybertsen, M. S. & Reichman, D. R. Theory of neutral and charged excitons in monolayer transition metal dichalcogenides. *Phys. Rev. B* **88**, 045318 (2013).

[5] Chernikov, A. *et al.* Exciton binding energy and nonhydrogenic Rydberg series in monolayer WS_2. *Phys. Rev. Lett.* **113**, 076802 (2014).

[6] Cudazzo, P., Tokatly, I. V. & Rubio, A. Dielectric screening in two-dimensional insulators: Implications for excitonic and impurity states in graphane. *Phys. Rev. B* **84**, 085406 (2011).

[7] Esser, A., Zimmermann, R. & Runge, E. Theory of trion spectra in semiconductor nanostructures. *physica status solidi (b)* **227**, 317–330 (2001).

[8] Deilmann, T., Drüppel, M. & Rohlfing, M. Three-particle correlation from a many-body perspective: Trions in a carbon nanotube. *Phys. Rev. Lett.* **116**, 196804 (2016).
